# Supplementary material for: Consultation-Based Deprescribing Service to Optimize Palliative Care for Terminal Cancer Patients
Source: J Clin Med. 2023 Nov 30;12(23):7431. doi: 10.3390/jcm12237431 (PMC10706958; doi:10.3390/jcm12237431)
Supplement: Supplementary file 1 [file jcm-12-07431-s001.zip › jcm-2730881-supplementary.pdf]

Supplementary data to the manuscript

Contents

Section	Title of the supplementary material
**Methods**	
Method S1	Development framework and process of pharmacist-led deprescribing service in consultation-based palliative care team (CB-PCT)
Method S2	Summary of the developed deprescribing guidelines for terminally ill patients with advanced cancer
**Tables**	
Table S1	Medication-related problems (MRPs) identified in patients cared by a consultation-based palliative care team (CB-PCT)
Table S2	Results of graph metrics of the sociogram analysis
Table S3	Frequency and acceptance rate (%) for each P/C code of medication-related problems (MRPs)
Table S4	Acceptance rate (%) of pharmacist's intervention by a drug class
Table S5	Factors associated with the occurrence of medication-related problems (MRPs)

Supplementary methods

S1. Development framework and process of pharmacist-led deprescribing service in consultation-based palliative care team (CB-PCT)

In the 'discover' phase, the aim was to identify unmet needs among patients, determine the target population and identify stakeholders for deprescribing service. Oncology pharmacists screened inpatients with limited life expectancy during a specific period, identifying the need for deprescribing. Group discussions about these needs helped shape the service setting and framework.

The subsequent 'define' phase focused on clarifying the core values, defining structure, process, and outcome, and creating a list of medications for the deprescribing guidelines. Group discussions and literature review were the main tool in define phase. The medication lists were derived not only from the previous studies [1–7], but also from the prescription reviews conducted by oncology pharmacists.

The 'design' phase began with the development of a deprescribing guideline, which involved conducting a systemic literature review for each medication class identified in the define phase. Once the draft guidelines were established, consensus meetings were organized to gather inputs and feedback. Subsequently, a service prototype was designed and further refined through prototyping and use case analysis. Moreover, department-specific forms were created to document and track pharmacist interventions, and these forms were integrated into the electronic medical record (EHR) system.

Finally, in the develop phase, service modeling and service blueprints were the primary tools used for formulating the final service model design and standardizing the service protocols.

Summary of each phase in 4D framework of the service design

Phase	Objectives of the process	Service tool used during the process
**Discover**	• Identifying the unmet needs	• Screening the inpatients with limited life expectancy over a certain period
	• Selecting the target population and multidisciplinary service providers	• Group discussion
**Define**	• Addressing the core values of the service	• Group discussion
	• Defining structure, process, and outcome of the service	• Group discussion
	• Obtaining a list of medications included in the guidelines	• Literature review
**Design**	• Developing a deprescribing guideline	• Group discussion<br>• Systematic literature review for deprescribing each medication class<br>• Consensus meeting for inputs and feedback on the draft guidelines
	• Designing a service prototype	• Prototyping
	• Improving the service prototype	• Consensus meeting for inputs and feedback on the service prototype
	• Creating department-specific formats for documenting and intervening pharmacist's services to be incorporated into the EHR	• Group discussion<br>• Use case analysis
**Develop**	• Formulating a final service model design	• Service modeling
	• Standardizing the service protocols	• Service blueprint

EHR, Electronic health record

Supplementary methods

S2. Summary of the developed deprescribing guidelines for terminally ill patients with advanced cancer

This guideline is designed for patients with advanced cancer whose life expectancy is projected to be less than 6 months or who possess an Eastern Cooperative Oncology Group (ECOG) performance status of 2 or higher.

Medication class	Medication	Considerations for deprescribing	Discontinuation / De-escalation recommendations	References
Antihypertensives	ACEI / ARBs<br>Beta blockers<br>CCB<br>Diuretics<br>Thiazide	• Used for secondary prevention of cardiovascular events or for the treatment of hypertension<br>• Duplicated use of ARBs and ACEIs<br>• Use of loop diuretics primarily for BP reduction<br>• SBP maintained <135 mmHg for the last 3 months	1. If one or more of the following conditions apply, decision to deprescribe is postponed.<br>- Currently comorbid angina<br>- History of cardiac arrest<br>- Comorbid heart failure<br>- History of myocardial infarction or cardiac revascularization within the past 3 years<br>- Suffered a stroke within the past 12 months<br>2. The target blood pressure is established as 150/90 mmHg.<br>3. Recommendations on priority discontinuation<br>- Discontinue one of the medications when ARBs and ACEIs are being used concurrently.<br>- Discontinue loop diuretics primarily prescribed for BP reduction.<br>4. Recommendations on stepwise de-escalation<br>- If the patient's SBP has maintained over the past 3 months, consider discontinuation of the antihypertensive drug that was added most recently.<br>- CCBs, ARBs, ACEIs, and thiazide diuretics can be discontinued immediately.<br>- For beta blockers, reduce the dosage by half for a week, and then completely discontinue in the following week.<br>- If any medications aside from the aforementioned (CCBs, ARBs, ACEIs, Thiazides, and Beta-blockers) are in use, identify potential antihypertensive drugs that could be discontinued and consider their discontinuation based on clinical judgment.<br>5. Recommendations on monitoring<br>- After discontinuing antihypertensive drugs, monitor the	[1–11]

			patient for symptoms related to blood pressure fluctuations such as orthostatic hypotension and dizziness.<br>- Assess the possibility for further discontinuation of medications.	
Hypoglycemic agents	Acarbose<br>DPP-4 inhibitors<br>GLP-1 analogues<br>Metformin<br>Sulfonylureas<br>Thiazolidinediones<br>SGLT2 inhibitors	• If the patient's glycated hemoglobin or random/fasting blood sugar is consistently below the target<br>• Patients taking more than two hypoglycemic agents for blood sugar control<br>• Use of hypoglycemic agents for mild hyperglycemia for secondary prevention of diabetic complications	1. For patients with a limited life expectancy, strict blood sugar control can lead to hypoglycemia, and discomfort from frequent subcutaneous injections can reduce the quality of life. Therefore, it is recommended to control blood sugar according to relaxed standards and it is important to confirm whether dietary management and self-blood sugar control are being performed well through consultation with the patient.<br>2. The target glycated hemoglobin (HbA1c) is established as less than 8.5%, and fasting blood glucose should be set at less than 200mg/dL.<br>- If a patient is undergoing hemodialysis, has had recent bleeding or a transfusion, or is receiving erythropoietin treatment which results in rapid red blood cell turnover, HbA1c may not accurately reflect blood sugar levels, thus the fasting blood glucose should be the primary target.<br>3. Deprescribing is considered based on the types of diabetes, and dietary feasibility of patients<br>- For type 1 DM patients and those requiring insulin due to pancreatic resection regardless of DM types, it is recommended to maintain the minimum basal insulin and premeal insulin (or mixed insulin) necessary for blood sugar control.<br>- For type 2 DM patients who are unable to eat orally and are on EN or TPN, it is recommended to stop all oral hypoglycemic agents (OHA), and intervene to control blood sugar with just basal insulin and premeal insulin.<br>- For type 2 DM patients who are able to eat orally and are on steroids, blood sugar levels may significantly increase upon	[1, 5, 7, 10, 12–15]

cessation of hypoglycemic agents, so continuous blood sugar monitoring is necessary and drug intervention should be considered when steroid medication is stopped in the future. However, if immediate drug discontinuation or dose reduction is needed due to worsening liver function or renal function, this should be reported to the medical team.

4. If the patient is taking an OHA that is contraindicated, drug discontinuation or change should be recommended primarily.
5. Recommendations on stepwise de-escalation for patients taking multiple hypoglycemic agents
   - If the HbA1c has been maintained at less than 7% (fasting blood glucose less than 155mg/dL) for the recent three months, or if the patient has experienced hypoglycemia symptoms at least once within the recent three months while maintaining an HbA1c of less than 7.5% (fasting blood glucose less than 170mg/dL), it is recommended to discontinue one hypoglycemic agent.
   - If the patient is administering a subcutaneous injectable hypoglycemic agent such as insulin or GLP-1 receptor agonists (exenatide, lixisenatide, dulaglutide, liraglutide), it is recommended to prioritize discontinuation of these over oral hypoglycemic agents.
   - Sulfonylureas carry a substantial risk of hypoglycemia, and this risk is further amplified when concurrently used with trimethoprim/sulfamethoxazole leading to increased serum concentrations. Therefore, among OHAs, discontinuation of sulfonylureas is recommended first.
   - Following sulfonylureas, meglitinides and α-glucosidase inhibitors carry a high risk of hypoglycemia. If the patient experiences frequent hypoglycemia symptoms, it is recommended to discontinue these medications
   - TZD drugs, when taken by patients with renal failure, can lead to fluid retention causing edema and increased risk of heart failure worsening. Thus, it is recommended to discontinue TZDs in patients with reduced renal function.
   - Selection of drug to intervene should be based on the patients' individual hypoglycemia experiences and current disease state. The fact that an 1% decrease in HbA1c is equivalent to a 29mg/dL decrease in blood glucose should be taken into

			consideration when assessing the degree of blood sugar rise upon drug discontinuation.<br>6. Recommendations on monitoring<br>- Monitor if the patients' blood sugar is controlled below the target values, and consider resuming the discontinued hypoglycemic agent after consulting with a healthcare professional.<br>- It is crucial not to overburden the patient with tests in order to monitor their blood sugar control.	
Lipid lowering agents	Ezetimibe<br>Fibrates<br>Statins	All indications	Discontinue medications	[1, 7, 16]
Antiplatelet agents	Aspirin<br>Cilostazol<br>Clopidogrel<br>Sarpogrelate	• For primary prevention<br>• For patients presenting severe thrombocytopenia associated with their diseases or liver dysfunction	Discontinue medications	[1]
Anticoagulants	DOACs<br>Warfarin	• Continuing drugs after completing treatment for DVT/PE<br>• For primary prevention of stroke in patients with atrial fibrillation<br>• For patients presenting severe thrombocytopenia associated with their diseases or liver dysfunction	1. Discontinue anticoagulants in the following cases<br>- In cases where provoking risk factors no longer persist, for patients who have been on treatment for DVT for more than 6 months or for pulmonary embolism (PE) for more than 12 months<br>- For patients who have severe thrombosis that requires further medical interventions such as transfusion<br>2. The risk of gastrointestinal bleeding should be monitored especially when NSAIDs are used in combination with vitamin K antagonists, direct thrombin inhibitors, or factor Xa inhibitors.	[1, 5, 17]

Osteoporosis medications	Bisphosphonates<br>Denosumab<br>Raloxifene	Used for the treatment and prevention of osteoporosis	Discontinue bisphosphonates unless they are used for the treatment of hypercalcemia secondary to bone metastases	[1, 7]
Pain managing agents	Acetaminophen*<br>Anticonvulsants*<br>Opioids<br>NSAIDs	• When the harm is greater than the risk<br>• When the efficacy of current drugs is not sufficient	1. Discontinue medications in the following cases<br>- Tramadol combined with other opioid analgesics<br>- Fentanyl prescribed for first-time user<br>- If multiple NSAIDs are used concurrently, all except one drug should be discontinued, due to an increase in adverse effects outweighing the benefits.<br>- TCAs are recommended to discontinue if patients do not tolerate adverse events, such as hear diseases, benign prostatic hyperplasia, neurogenic bladder, dementia, and narrow-angle glaucoma which may be worsened by TCAs in geriatric populations.<br>2. Recommendations on the optimal use of pain managing agents<br>- Switch patients from pethidine to other opioid analgesics due to the risk of seizures or arrhythmias from accumulation of its metabolic product.<br>- Nalbuphine and buprenorphine used for patients currently taking pure opioids, should be cautioned due to the risk of withdrawal symptoms.<br>- Avoid the use of indomethacin and ketorolac in geriatric patients due to the high risk of GI bleeding, peptic ulcers, and renal impairment<br>- Ensure that patients taking opioids are prescribed stimulant and osmotic laxatives to prevent constipation.	[14, 18]
Gastroprotective agents	H_2 antagonists<br>PPIs<br>Mucosal protectors	• Lack of history of GI diseases diagnosed via endoscopy, GI bleeding, peptic ulcers, gastritis, GERD, or those who are not taking NSAIDs or steroids for more than 30 days<br>• Long-term use without proper indication	1. Recommendations on deprescribing<br>- Stepwise de-escalation is recommended for gastric acid secretion inhibitors due to rebound syndrome. (e.g. reduce dose by 50% every 1–2 weeks, stop the drug once at 25% of the original dose and no withdrawal symptoms have been observed)<br>2. Specific recommendations on each type of medications<br>- PPIs used for more than 8 weeks should be avoided as they increase the risk of *Clostridium difficile* infection, osteoporosis, and fracture.<br>- Long term use of PPI without indication should be changed to	[1, 7, 14, 19]

		identified	another class of medication, unless the patients are failed in attempts to discontinue or switch to H2 antagonists.<br>- H2 receptor antagonists should be avoided in patients with delirium or with high risk of delirium.	
Prokitenic agents	D_2 antagonist<br>Serotonergic agonist	Long term use without proper indication identified	1. If one or more of the following conditions apply, decision to deprescribe is postponed.<br>- Persisting nausea or vomiting due to chemotherapy<br>- Existing gastroparesis<br>2. Otherwise, consider discontinue medications.	[14]
Parasympatho-mimetics	Acetyl-L-carnitine<br>Choline alfoscerate<br>Oxiracetam	All indications	Discontinue medication	[20]
Complemantary-alternative medicine	Vitamins<br>Minerals<br>Probiotics<br>Ursodiol<br>Silymarin	Preventive use of medications	1. If one or more of the following conditions apply, decision to deprescribe is postponed.<br>- When the medications are used to correct low blood plasma concentrations, based on clinical judgements<br>2. Otherwise, discontinue medications.	[1]

*Only when the medication is used for pain management

ACEI, Angiotensin-converting enzyme inhibitor; ARB, angiotensin receptor blockers; BP, blood pressure; CCB, calcium channel blocker; DOAC, direct-acting oral anticoagulants; DM, diabetes mellitus; DPP-4 inhibitor. dipeptidyl peptidase-4 inhibitor; DVT, deep vein thrombosis; EN, enteral nutrition; GERD, gastroesophageal reflux disease; GI, gastrointestinal; GLP-1 analogue, Glucagon-like peptide-1 analogue; NSAID, Non-steroidal anti-inflammatory drug; OHA, oral hypoglycemic agent; PE, pulmonary embolism; PPI, proton pump inhibitor; SGLT2 inhibitor, Sodium-glucose cotransporter-2 Inhibitor; TCA, tri-cyclic antidepressant; TPN, total parenteral nutrition; TZD, thiazolidinedione;

1. Lindsay J, et al. The development and evaluation of an oncological palliative care deprescribing guideline: the 'OncPal deprescribing guideline'. Support Care Cancer. 2015;23(1):71–8.
2. Sheppard JP, et al. OPtimising Treatment for MIld Systolic hypertension in the Elderly (OPTiMISE): protocol for a randomised controlled non-inferiority trial. BMJ Open. 2018;8(9):e022930.
3. Moonen JE, et al. Effect of discontinuation of antihypertensive medication on orthostatic hypotension in older persons with mild cognitive impairment: the DANTE Study Leiden. Age Ageing. 2016;45(2):249-55.
4. Whelton PK, et al. 2017 ACC/AHA/AAPA/ABC/ACPM/AGS/APhA/ASH/ASPC/NMA/PCNA Guideline for the Prevention, Detection, Evaluation, and Management of High Blood Pressure in Adults: Executive Summary: A Report of the American College of Cardiology/American Heart Association Task Force on Clinical Practice Guidelines. J Am Coll Cardiol. 2018;71(19):2199-269.
5. O'Mahony D, et al. STOPP/START criteria for potentially inappropriate prescribing in older people: version 2. Age Ageing. 2015;44(2):213-8.
6. Williamson JD, et al. Intensive vs Standard Blood Pressure Control and Cardiovascular Disease Outcomes in Adults Aged ≥75 Years: A Randomized Clinical Trial. Jama. 2016;315(24):2673–82.
7. Lavan AH, et al. STOPPFrail (Screening Tool of Older Persons Prescriptions in Frail adults with limited life expectancy): consensus validation. Age Ageing.

2017;46(4):600–7.
8. Aspinall SL, et al. Deprescribing in older nursing home patients: Focus on innovative composite measures for dosage deintensification. Innov Aging. 2017;1(2):igx031.
9. James PA, et al. 2014 evidence-based guideline for the management of high blood pressure in adults: report from the panel members appointed to the Eighth Joint National Committee (JNC 8). JAMA. 2014;311(5):507–20.
10. American Diabetic Association (ADA). 12. Older Adults: Standards of Medical Care in Diabetes-2019. Diabetes Care. 2019;42(Suppl 1):S139–47.
11. Shin J, Cho MC. Updated reasons and clinical implications of bew Korean hypertension guidelines for cardiologists. Korean Circ J. 2020;50(6):476–84.
12. Farrell B, et al. Deprescribing antihyperglycemic agents in older persons: Evidence-based clinical practice guideline. Can Fam Physician. 2017;63(11):832–43.
13. Lee JM. Antihyperglycemic agent combination therapy for patients with type 2 diabetes mellitus. J Korean Med Assoc. 2014;57(5):435–43.
14. American Geriatrics Society 2019 Updated AGS Beers Criteria® for Potentially Inappropriate Medication use in older adults. J Am Geriatr Soc. 2019 Apr;67(4):674–94.
15. Kim MK, et al. 2019 Clinical practice guidelines for type 2 diabetes mellitus in Korea. Diabetes Metab J. 2019;43(4):398–406.
16. Lindsay J, et al. Reducing potentially inappropriate medications in palliative cancer patients: evidence to support deprescribing approaches. Support Care Cancer. 2014;22(4):1113–9.
17. Jung KH, et al. Antithrombotic management of patients with nonvalvular atrial fibrillation and ischemic stroke or transient ischemic attack: Executive summary of the Korean clinical practice guidelines for stroke. J Stroke. 2015;17(2):210–5.
18. Swarm RA, et al. Adult Cancer Pain, Version 3.2019, NCCN Clinical Practice Guidelines in Oncology. J Natl Compr Canc Netw. 2019;17(8):977–1007.
19. Vrijkorte E, et al. Optimising pharmacotherapy in older cancer patients with polypharmacy. Eur J Cancer Care (Engl). 2020;29(1):e13185.
20. Renom-Guiteras A, et al. The EU(7)-PIM list: a list of potentially inappropriate medications for older people consented by experts from seven European countries. Eur J Clin Pharmacol. 2015;71(7):861–75.

Supplementary methods

References

1. Lindsay J, Dooley M, Martin J, et al. The development and evaluation of an oncological palliative care deprescribing guideline: the 'OncPal deprescribing guideline'. Support Care Cancer. 2015;23(1):71–8.
2. Fede A, Miranda M, Antonangelo D, et al. Use of unnecessary medications by patients with advanced cancer: cross-sectional survey. Support Care Cancer. 2011;19(9):1313–8.
3. Whitman A, DeGregory K, Morris A, Mohile S, Ramsdale E. Pharmacist-led medication assessment and deprescribing intervention for older adults with cancer and polypharmacy: a pilot study. Support Care Cancer. 2018;26(12):4105–13.
4. Alves A, Green S, James DH. Deprescribing of Medicines in Care Homes-A Five-Year Evaluation of Primary Care Pharmacist Practices. Pharmacy (Basel). 2019;7(3):105.
5. Baqir W, Hughes J, Jones T, et al. Impact of medication review, within a shared decision-making framework, on deprescribing in people living in care homes. Eur J Hosp Pharm. 2017;24(1):30–3.
6. Beer C, Loh PK, Peng YG, Potter K, Millar A. A pilot randomized controlled trial of deprescribing. Ther Adv Drug Saf. 2011;2(2):37–43.
7. Oliveira L, Ferreira MO, Rola A, Magalhães M, Ferraz Gonçalves J. Deprescription in Advanced Cancer Patients Referred to Palliative Care. J Pain Palliat Care Pharmacother. 2016;30(3):201–5.

Supplementary Tables

Table S1. Medication-related problems (MRPs) identified in patients cared by a consultation-based palliative care team (CB-PCT)

Problem domains	Sub-domains	UC group (N (%)) (N = 16)	AC group (N (%)) (N = 200)	Total (N (%)) (N-216)
**P1. Treatment effectiveness**	P1.1 No effect of drug treatment despite correct use	2 (12.5)	3 (5.5)	5 (2.31)
	P1.2 Effect of drug treatment not optimal	1 (6.2)	36 (18)	37 (17.13)
	P1.3 Untreated symptoms or indication	1 (6.2)	6 (3)	7 (3.24)
**P2. Treatment safety**	P2.1 Adverse drug event (possibly) occurring	2 (12.5)	51 (25.5)	53 (24.54)
**P3. Other**	P3.1 Unnecessary drug-treatment	6 (12.5)	93 (46.5)	99 (45.83)
	P3.2 Unclear problem / complaint	4 (25.0)	11 (5.5)	15 (6.94)
	Total			*216 (100)*

Cause domains	Sub-domains	UC group (N (%)) (N=16)	AC group (N (%)) (N=200)	Total (N (%)) (N=216)
**C1. Drug selection**	C1.1 Inappropriate drug according to guidelines/formulary	0 (0.0)	34 (17.0)	34 (15.7)
	C1.2 No indication for drug	3 (18.8)	88 (44.0)	91 (42.1)
	C1.3 Inappropriate combination of drugs, or drugs and herbal medications, or drugs and dietary supplements	0 (0.0)	13 (6.5)	13 (6.02)
	C1.4 Inappropriate duplication of therapeutic group or active ingredient	0 (0.0)	2 (1.0)	2 (0.92)
	C1.5 No or incomplete drug treatment in spite of existing indication	2 (12.5)	15 (7.5)	17 (7.87)
	C1.6 Too many different drugs/active ingredients prescribed for indications	2 (12.5)	3 (1.5)	5 (2.31)
**C2. Drug form**	C2.1 Inappropriate drug forms/formulation	0 (0.0)	0 (0.0)	0 (0.0)
**C3. Drug selection**	C3.1 Drug dose too low	1 (6.2)	5 (2.5)	6 (2.78)
	C3.2 Drug dose of a single active ingredient	0 (0.0)	8 (4.0)	8 (3.70)

	C3.3 Dosage regimen not frequent enough	1 (6.2)	2 (1.0)	3 (1.39)
	C3.4 Dosage regimen too frequent	0 (0.0)	7 (3.5)	7 (3.24)
	C3.5 Dose timing instructions wrong, unclear or missing	0 (0.0)	0 (0.0)	0 (0.0)
**C4. Treatment duration**	C4.1 Duration of treatment too short	0 (0.0)	0 (0.0)	0 (0.0)
	C4.2 Duration of treatment too long	0 (0.0)	0 (0.0)	0 (0.0)
**C5. Dispensing**	C5.1 Prescribed drug not available	1 (6.2)	0 (0.0)	1 (0.46)
	C5.2 Necessary information not provided or incorrect advice provided	0 (0.0)	0 (0.0)	0 (0.0)
	C5.3 Wrong drug, strength or dosage advised	0 (0.0)	0 (0.0)	0 (0.0)
	C5.4 Wrong drug or strength dispensed	0 (0.0)	0 (0.0)	0 (0.0)
**C6. Drug use process**	C6.1 Inappropriate timing of administration or dosing intervals by a health professional	0 (0.0)	0 (0.0)	0 (0.0)
	C6.2 Drug under-administered by a health professional	0 (0.0)	0 (0.0)	0 (0.0)
	C6.3 Drug over-administered by a health professional	2 (12.5)	0 (0.0)	2 (0.92)
	C6.4 Drug not administered at all by a health professional	0 (0.0)	0 (0.0)	0 (0.0)
	C6.5 Wrong drug administered by a health professional	0 (0.0)	0 (0.0)	0 (0.0)
	C6.6 Drug administered via wrong route by a health professional	0 (0.0)	0 (0.0)	0 (0.0)
**C7. Patient related**	C7.1 Patient intentionally uses/takes less drug than prescribed or does not take the drug at all for whatever reason	0 (0.0)	2 (1.0)	2 (0.92)
	C7.2 Patient uses/takes more drug than prescribed	0 (0.0)	0 (0.0)	0 (0.0)
	C7.3 Patient abuses drug (unregulated overuse)	0 (0.0)	0 (0.0)	0 (0.0)
	C7.4 Patient decides to use unnecessary drug	0 (0.0)	0 (0.0)	0 (0.0)
	C7.5 Patient takes food that interacts	0 (0.0)	0 (0.0)	0 (0.0)
	C7.6 Patient stores drug inappropriately	0 (0.0)	0 (0.0)	0 (0.0)

	C7.7 Inappropriate timing or dosing intervals	0 (0.0)	0 (0.0)	0 (0.0)
	C7.8 Patient unintentionally administers/uses the drug in a wrong way	0 (0.0)	0 (0.0)	0 (0.0)
	C7.9 Patient physically unable to use drug/form as directed	0 (0.0)	7 (3.5)	7 (3.24)
	C7.10 Patient unable to understand instructions properly	0 (0.0)	0 (0.0)	0 (0.0)
**C8. Patient transfer related**	C8.1 Medication reconciliation problem	0 (0.0)	1 (0.5)	1 (0.46)
**C9. Other**	C9.1 No or inappropriate outcome monitoring	0 (0.0)	1 (0.5)	1 (0.46)
	C9.2 Other cause	0 (0.0)	0 (0.0)	0 (0.0)
	C9.3 No obvious cause	0 (0.0)	1 (0.5)	1 (0.46)

AC, active care; UC, usual care;

Supplementary Table S2. Results of graph metrics of the sociogram analysis

Vertices	Degree centrality		Betweenness Centrality	Closeness Centrality
	In-degree (C_{D-in})	Out-degree (C_{D-out})		
**Major causative agents**				
Pain managing agents	-	8	144.937	0.012
Drugs for urinary frequency and incontinence	-	6	88.810	0.011
Gastroprotective agents	-	6	58.116	0.012
Antibiotics	-	6	53.271	0.011
Liver supplements	-	4	18.354	0.011
Lipid lowering agents	-	4	14.986	0.011
Complementary therapies	-	4	25.083	0.011
Osteoporosis medications	-	4	33.940	0.011
Neuropsychiatric drugs	-	4	14.986	0.011
Antihypertensives	-	3	9.676	0.010
Hypoglycemic agents	-	3	64.386	0.011
Antiemetics	-	3	24.743	0.011
Chemotherapeutic agents	-	3	13.107	0.010
Vitamins and minerals	-	2	17.085	0.009
Antiplatelet agents	-	2	3.495	0.010
Prokinetics	-	2	2.527	0.010
Parasympathomimetics	-	1	0.000	0.009
Immunosuppressants	-	1	0.000	0.007
**Cause domains**				
C1.2. No indication for drug	15	1	447.779	0.014
C1.1. Inappropriate drug according to guidelines/formulary	11	3	317.051	0.013
C1.3. Inappropriate combination of drugs, or drugs and herbal medications, or drugs and dietary supplements	7	2	96.883	0.011
C1.5. No or incomplete drug treatment in spite of existing indication	4	4	158.511	0.011
C7.9. Patient physically unable to use drug/form as directed	5	2	73.193	0.011
C3.2. Drug dose of a single active ingredient too high	4	2	47.776	0.010
C1.6. Too many different drugs/active ingredients prescribed for indication	4	1	31.388	0.010
C3.3. Dosage regimen not frequent enough	3	2	29.151	0.009
C3.1. Drug dose too low	3	2	93.971	0.009
C7.1. Patient intentionally uses/takes less drug than prescribed or does not take the drug at all for whatever reason	2	2	90.187	0.009
C3.4. Dosage regimen too frequent	2	2	12.748	0.010
C6.3. Drug over-administered by a health professional	2	1	11.471	0.009
C1.4. Inappropriate duplication of therapeutic group or active ingredient	1	2	8.337	0.009
C5.1. Prescribed drug not available	1	1	3.347	0.008
C9.3. No obvious cause	1	1	2.372	0.008

C9.1. No or inappropriate outcome monitoring	1	1	2.636	0.009
C8.1. Medication reconciliation problem	1	1	7.198	0.008
**Problem domains**				
P2.1. Adverse drug event (possibly) occurring	10	-	236.084	0.012
P1.2. Effect of drug treatment not optimal	9	-	149.451	0.012
P3.1. Unnecessary drug-treatment	5	-	100.850	0.011
P1.1. No effect of drug treatment despite correct use	4	-	15.819	0.008
P1.3. Untreated symptoms or indication	2	-	18.197	0.008

Supplementary Table S3. Frequency and acceptance rate (%) for each P/C code of medication-related problems (MRPs)

P/C code		Count	Acceptance rate (%)
P3.1/C1.2	(Unnecessary drug-treatment / No indication for drug)	91	9.9
P2.1/C1.1	(Adverse drug event (possibly) occurring / Inappropriate drug according to guidelines/formulary)	23	26.1
P3.2/C9.3	(Unclear problem / No obvious cause)	15	100
P1.2/C1.1	(Effect of drug treatment not optimal / Inappropriate drug according to guidelines/formulary)	10	30
P2.1/C1.3	(Adverse drug event (possibly) occurring / Inappropriate combination of drugs, or drugs and herbal medications, or drugs and dietary supplements)	8	50
P1.2/C1.5	(Effect of drug treatment not optimal / No or incomplete drug treatment in spite of existing indication)	7	28.6
P1.3/C1.5	(Untreated symptoms or indication / No or incomplete drug treatment in spite of existing indication)	6	66.7
P3.1/C1.6	(Unnecessary drug-treatment / Too many different drugs/active ingredients prescribed for indication)	5	80
P2.1/C3.4	(Adverse drug event (possibly) occurring / Dosage regimen too frequent)	5	60
P2.1/C7.9	(Adverse drug event (possibly) occurring / Patient physically unable to use drug/form as directed)	5	60
P1.2/C1.3	(Effect of drug treatment not optimal / Inappropriate combination of drugs, or drugs and herbal medications, or drugs and dietary supplements)	5	40
P1.2/C3.2	(Effect of drug treatment not optimal / Drug dose of a single active ingredient too high)	4	75
P2.1/C3.2	(Adverse drug event (possibly) occurring / Drug dose of a single active ingredient too high)	4	50
P1.2/C3.1	(Effect of drug treatment not optimal / Drug dose too low)	4	25
P2.1/C1.5	(Adverse drug event (possibly) occurring / No or incomplete drug treatment in spite of existing indication)	3	66.7
P1.1/C3.1	(No effect of drug treatment despite correct use / Drug dose too low)	2	100
P1.2/C3.4	(Effect of drug treatment not optimal / Dosage regimen too frequent)	2	100
P1.2/C3.3	(Effect of drug treatment not optimal / Dosage regimen not frequent enough)	2	50
P1.2/C7.9	(Effect of drug treatment not optimal / Patient physically unable to use drug/form as directed)	2	50
P2.1/C6.3	(Adverse drug event (possibly) occurring / Drug over-administered by a health professional)	2	50
P1.1/C1.4	(No effect of drug treatment despite correct use / Inappropriate duplication of therapeutic group or active ingredient)	1	100
P1.1/C1.5	(No effect of drug treatment despite correct use/ No or incomplete drug treatment in spite of existing indication)	1	100
P1.1/C3.3	(No effect of drug treatment despite correct use / Dosage regimen not frequent enough)	1	100
P1.2/C1.4	(Effect of drug treatment not optimal / Inappropriate duplication of therapeutic group or active ingredient)	1	100
P1.3/C8.1	(Untreated symptoms or indication / Medication reconciliation problem)	1	100
P2.1/C7.1	(Adverse drug event (possibly) occurring / Patient intentionally uses/takes less drug than prescribed or does not take the drug at all for whatever reason)	1	100
P2.1/C9.1	(Adverse drug event (possibly) occurring / No or inappropriate outcome monitoring (incl. TDM))	1	100

P2.1/C9.3	(Adverse drug event (possibly) occurring / No obvious cause)	1	100
P3.1/C1.1	(Unnecessary drug-treatment / Inappropriate drug according to guidelines/formulary)	1	100
P3.1/C5.1	(Unnecessary drug-treatment / Prescribed drug not available)	1	100
P3.1/C7.1	(Unnecessary drug-treatment / Patient intentionally uses/takes less drug than prescribed or does not take the drug at all for whatever reason)	1	100

Table S4. Acceptance rate (%) of pharmacist's intervention by a drug class

Drug class	Usual care (UC)		Active care (AC)	
	**N (%)**	**Acceptance rate (%)**	**N (%)**	**Acceptance rate (%)**
Pain managing agents	5 (31.3)	100	40 (20)	92.5
Lipid lowering agents	1 (6.3)	0	25 (12.5)	72
Gastroprotective agents	0 (0)	-	21 (10.5)	81.0
Complementary therapies	1 (6.3)	0	17 (8.5)	82.4
Hypoglycemic agents	0 (0)	-	13 (6.5)	58.3
Vitamins and minerals	2 (12.5)	100	12 (6.0)	91.7
Liver supplements	2 (12.5)	50	12 (6.0)	66.7
Anti-infectives	0 (0)	-	11 (5.5)	54.6
Antihypertensives	0 (0)	-	10 (10)	90
Neuropsychiatric drugs	0 (0)	-	9 (4.5)	66.7
Drugs for urinary frequency and incontinence	1 (6.3)	100	7 (3.5)	85.7
Prokinetics	0 (0)	-	6 (3.0)	66.7
Antiplatelet agents	0 (0)	-	4 (2.0)	75
Osteoporosis medications	2 (12.5)	0	4 (2.0)	75
Chemotherapeutic agents	1 (6.3)	100	3 (1.5)	33.3
Antiemetics	1 (6.3)	100	3 (1.5)	100
Parasympathomimetics	0 (0)	-	1 (0.5)	100
Hormones	0 (0)	-	1 (0.5)	100
Immunosuppressants	0 (0)	-	1 (0.5)	100
Medications to treat cardiovascular diseases (except for antihypertensives)	0 (0)	-	0 (0)	-
Anticoagulants	0 (0)	-	0 (0)	-

Supplementary table S5. Factors associated with the occurrence of medication-related problems (MRPs)

Factor	Univariate analysis		Multivariate analysis*	
	**OR (95% CI)**	***P*-value**	**Adjusted OR (95% CI)**	***P*-value**
Hypertension	2.38 (1.35–4.21)	<0.01	-	-
Diabetes	2.63 (1.44–4.8)	<0.01	2.15 (1.12–4.13)	0.02
Ischemic heart disease	2.53 (1.47–4.38)	<0.01	1.85 (1.01–3.39)	0.04
Continuation of cancer treatment	1.74 (0.99–3.04)	0.05	1.66 (0.96–2.7)	0.06
Number of oral medications	1.04 (1.02–1.07)	<0.01	-	-
High pill burden†	5.54 (3.3–9.25)	<0.01	4.51 (2.61–7.8)	<0.01
Polypharmacy[a]	4.25 (1.66–10.89)	<0.01	-	-
Newly diagnosis of cancer	3.72 (1.20–11.52)	0.02	4.22 (1.23–14.4)	0.02

OR, Odds ratio

*Reduced model was used

†High pill burden was defined as the use of five or more oral medications.

[a]Polypharmacy was defined as the use of five or more medications in total.
